# Exercise Effects on Cognition in Older African Americans: A Pilot Randomized Trial

**DOI:** 10.3389/fnagi.2022.921978

**Published:** 2022-07-22

**Authors:** Kathryn L. Gwizdala, Robert Brouillete, Robbie Beyl, William Johnson, Callie Hebert, Leah Carter, Melissa Harris, Robert L. Newton, Owen T. Carmichael

**Affiliations:** Pennington Biomedical Research Center, Baton Rouge, LA, United States

**Keywords:** physical activity, Alzheimer’s disease, cognitive function, African American, dementia prevention

## Abstract

**Introduction:**

Regular physical activity lowers risk for cognitive decline and neurodegenerative disorders. Older African Americans (AAs) have been underrepresented in trials that increased physical activity to improve cognitive outcomes.

**Methods:**

56 sedentary, older, cognitively healthy AAs (avg. 69.2 ± 3.4 yrs. old) were randomized in 1:1 ratio into either a 12-week successful aging group (SAG) or a 12-week physical activity group (PAG). Participants in SAG attended weekly 60-min educational sessions in which healthy aging topics were discussed. Participants in PAG attended supervised physical activity sessions twice per week at local YMCAs (90–120 min/week) and were prescribed 2–3 days per week of home-based activity. The Repeatable Battery for the Assessment of Neuropsychological Status (RBANS) assessed cognitive function. ANCOVA models compared mean 12-week change in global cognition and subdomain scores between groups with secondary analyses for sex differences. Effect sizes for RBANS were calculated.

**Results:**

The RBANS global cognition score (SAG Est. 5.6 ± 1.8, effect size = 0.37, *p* = 0.003) and several subdomain scores (one-sample *T* tests, all *p* < 0.05) increased significantly within the SAG. Scores for global cognition increased more in SAG than in PAG (Change Estimate, PAG minus SAG: –4.6 ± 2.5 points, effect size = 0.31) at a trend level (*p* = 0.072). SAG females increased their global cognition score more than PAG females and more than males in either PAG or SAG (all *p* < 0.035).

**Discussion:**

A 12-week physical activity intervention (PAG) did not improve cognitive functioning among older AAs but a comparator healthy aging education program did. Inadequate physical activity dosage or duration, SAG members acting on health-related information from educational sessions, and/or social stimulation within the SAG may have contributed to these results. Future studies should combine socially engaging activities with vigorous physical activity for cognitive enhancement among cognitively healthy older African Americans.

**Clinical Trial Registration:**

www.ClinicalTrials.gov, identifier NCT03474302.

## Highlights

- African Americans are underrepresented in health and aging research.
- One of few physical activity cognition interventions in older African Americans.
- Successful aging group improved cognition more than the physical activity group.
- Women showed greater benefits from the successful aging group.

## Introduction

The prevalence of Alzheimer’s Disease (AD) is steadily increasing in the general population with the number of cases projected to reach 14 million by 2060 (Matthews et al., 2018). African Americans (AAs) are two times more likely to have Alzheimer’s Disease or other dementias in comparison to whites (Barnes and Bennett, 2014; Alzheimer’s Association, 2021). They are also at a disproportionally high risk for metabolic diseases (e.g., cardiovascular disease, diabetes, obesity, etc.) that are associated with the development of Alzheimer’s Disease (Samper-Ternent and Al Snih, 2012; Mayeda et al., 2014; Tini et al., 2020). The high prevalence of dementia and its risk factors among AAs indicate a need for interventions targeting this population that are designed to reduce risk factors for developing dementia.

A growing body of literature suggests that physical activity may reduce risk of dementia (Meng et al., 2020). Cross sectional and prospective studies have shown that greater physical activity is associated with lower risk of cognitive decline (Blondell et al., 2014). Both short and long-term clinical trials suggest that increasing physical activity results in improvements in global and domain-specific cognitive function (Ngandu et al., 2015; Vidoni et al., 2015; Pontifex et al., 2016). Increasing physical activity may exert its effects on cognition by provoking metabolic and structural brain changes such as increasing brain volume, decreasing neuroinflammation, improving cerebral glucose metabolism, and enhancing functional connectivity (Colbert et al., 2004; Colcombe et al., 2006; Voss et al., 2010; Dougherty et al., 2017).

One limitation of the evidence base for cognitive benefits of physical activity interventions is underrepresentation of older AAs. This underrepresentation is present despite the fact that physical activity interventions could be particularly beneficial in this community. AAs have consistently reported levels of physical activity that are below national recommendations, and significantly lower than that of white Americans (Whitt-Glover et al., 2007; Troiano et al., 2008). In addition, current evidence of cognitive benefits of physical activity programs, gathered primarily from predominantly white cohorts, may not generalize directly to the African American population due to differences in exercise responses and biomarkers of aging (e.g., APOE4 genotype and telomere length) compared to corresponding white Americans (Swift et al., 2013; Brown et al., 2017; Yu et al., 2017). For example, isolated studies suggest that AA women’s cardiorespiratory fitness improvements attenuate at a lower dose of exercise compared to non-Hispanic white women (Swift et al., 2013) and that aerobic and strength training have shown differential effects on insulin resistance in AAs and white Americans (Winnick et al., 2008). In addition, a higher percentage of AAs are APOE4 carriers (Beydoun et al., 2021), which would serve to blunt neurotrophic responses to exercise (Allard et al., 2017). Finally, AAs experience more adverse social determinants of health (e.g., racial discrimination, residential segregation, neighborhood walkability) which can in turn affect treatment engagement and response. Only a small number of physical activity interventions have specifically targeted AAs (Lukach et al., 2016; Zhang and Jemmott, 2019; Fausto et al., 2021). None of the prior interventions, to our knowledge, followed designs informed by and tailored to older AAs in conjunction with utilizing objective measures of physical activity. In addition, none of the prior AA-targeted interventions assessed sex differences in intervention responses, despite evidence for such differences (Liu-Ambrose et al., 2018; Watts et al., 2018). Women and men have been found to have differing physiological and cognitive outcomes in physical activity interventions (Colcombe and Kramer, 2003; Barha and Liu-Ambrose, 2018; Arciero et al., 2022). The Program for African American Cognition and Exercise (PAACE) was designed to assess the effect of a physical activity intervention on cognitive function among cognitively healthy older AA men and women to address these research gaps.

## Materials and Methods

### Focus Groups

Focus groups with older African American adults with content related to dementia and willingness to participate in a physical activity intervention were conducted to inform the study design. More details about the focus groups and outcomes can be found in a prior publication (Pugh et al., 2021b). Briefly, the findings guided the study design toward an intervention located within the community, personalized to health conditions of the participants, and containing fun activities such as line dancing.

### Participants

Eligibility criteria included being 65–85 years old, self-identifying as African American, being classified as sedentary or insufficiently active (steps per day < 75th percentile for age and gender) (Tudor-Locke and Schuna, 2012), being free of cognitive impairment (Mini-Mental State Exam, MMSE, score > 26; O’Bryant et al., 2008), being free of chronic health conditions that would prevent study completion, being willing to participate in group sessions, and planning to live in the study area for at least the next 6 upcoming months. Participants were excluded from the study if they were unable or unwilling to give consent, had uncontrolled hypertension (systolic blood pressure > 200 mmHg and/or diastolic blood pressure > 110 mmHg), had a significant health condition that the principal investigator or medical investigator deemed to interfere with participation (e.g., hip fracture, hip or knee replacement, spinal surgery in the past 6 months, myocardial infarction, major heart surgery), or were enrolled in a different randomized trial focused on lifestyle or pharmaceutical intervention. The study was approved by the Pennington Biomedical Research Center Institutional Review Board and written informed consent was obtained from all participants.

### Study Design

Potential participants were initially screened over the telephone. Eligible participants were invited to attend an in-person orientation to learn about the study procedures in detail. Interested participants then signed informed consent, completed questionnaires regarding physical activity and demographics and were given an activity monitor (ActiGraph GT3X+ and Fitbit Charge 2^®^) to wear for 7 days. The participants returned for their baseline visit after 7 days. Their ActiGraph was initially assessed to determine whether they met inclusion criteria and eligible participants then completed baseline cognitive assessments and were randomized into the Successful Aging Group (SAG) or Physical Activity Group (PAG). Following the conclusion of the 12-week intervention, participants completed a follow up visit consisting of the same assessment battery that were completed at baseline. The study is registered in clinicaltrials.gov (NCT03474302).

### Intervention

Random assignment of participants was executed in a 1:1 ratio to either study group with the utilization of SAS, by the study statistician, to create covariate adaptive randomization (Hu and Hu, 2012). More details are published elsewhere (Newton et al., 2022).

#### Successful Aging Education

Participants attended weekly 30–60-min group sessions over 12 weeks. They received information on topics relevant to an older population, such as healthy eating, living wills, and dementia awareness. These groups provided a didactic component and fostered discussion among participants. Approximately 70% of the session was didactic and 30% was discussion. The successful aging education (SAG) sessions were conducted at the academic institution. Sessions were led by an individual with a Master’s degree in dietetics.

#### Physical Activity Intervention

The goal of the program was for participants to achieve 150 min of moderate to vigorous physical activity (MVPA) and 2 days of strength training per week [consistent with the current national recommendation at the time the study was funded (U.S. Department of Health and Human Services, 2008) as well as the most recent guidelines (Piercy et al., 2018)] through a combination of supervised and home-based activity. Two group-based supervised PA sessions per week were conducted by research staff at a local YMCA. The particular YMCA was chosen because it was located in a part of the city that is heavily populated with African American adults. Each session lasted approximately 45–60 min and consisted of aerobic, strength, balance, and flexibility training. Walking at a moderate intensity pace was the primary aerobic activity, but other aerobic activities, such as line-dancing, were incorporated. In addition, each participant progressively increased the intensity and duration of strength and balance exercises until the top level (three strength levels and five balance levels) in each category was reached. Two to four days of home-based PA was also prescribed. Participants were instructed to engage in ∼30 min walking bouts at home and to engage in strength, flexibility, and balance exercises at home that were provided *via* prescription cards. The target rate of perceived exertion (RPE, Borg scale) range for aerobic and strength exercises was 11–15. Sessions were led by individuals with Master’s degrees in exercise physiology.

### Measures

All measurements were taken at baseline and the 12-week follow up unless otherwise noted. All assessment personnel were blinded to the participant randomization.

#### Accelerometry

The Actigraph GT3X+ accelerometer (Actigraph LLC, Pensacola, FL) was utilized to measure step counts over the course of 7 days. Sedentary activity was defined as ≤ 100 activity counts per minute. Predetermined cut points were used to identify time in intensity of physical activity (i.e., light ≤ 1,951 counts/min^–1^, moderate 1,952–5,724 counts/min^–1^, hard 5,725–9,498 counts/min^–1^, and very hard intensity levels ≥ 9,499 counts/min^–1^) (Freedson et al., 1998).

#### Body Mass Index (Baseline Only)

Height and weight were obtained to calculate body mass index (BMI) (kg/m^2^). Weight was measured with a balance beam scale and measurements were taken to the nearest 0.5 kg. Height was measured with a standard stadiometer and measurements were taken to the nearest 0.1 cm.

#### Blood Draw

A fasting blood draw was obtained to measure fasting blood glucose, cholesterol, low-density lipoprotein (LDL) and high-density lipoprotein (HDL) levels.

#### Medication History

Participants were asked to report medications that they were currently taking or had taken within the last 30 days at the baseline visit. At the 12-week follow up, participants were asked if they had started any new medications or changes in medications since the baseline visit. Name, dose, frequency, route of administration, indication, and start and end date were all obtained for each medication reported.

#### Repeatable Battery for the Assessment of Neuropsychological Status

The Repeatable Battery for the Assessment of Neuropsychological Status (RBANS) is a comprehensive assessment of cognitive function (Randolph et al., 1998). The battery provides a measure of global cognitive function and subdomains including immediate memory (list learning and story memory), visuospatial function (figure copy and line orientation), language capacity (picture naming and semantic fluency), attention (digit span and coding), and delayed memory (list recall, list recognition, story recall and figure recall). The standardized global and subdomain scores have an average score of 100 units and a standard deviation of 15 units.

## Statistical Analysis

### Primary Analyses

Descriptive statistics were summarized as means and frequencies. A linear statistical model with repeated assessment of outcomes over time was used to estimate influences of treatment on change in cognitive performance. Variations in treatment differences across time were investigated as treatment by time interaction. An unstructured covariance matrix was employed in this repeated measurement model to account for correlations over time. This analytical model was used to produce estimates of least squares means outcomes relevant to differential treatment effects on changes in cognitive performance. The statistical significance of differential effects on cognitive performance was summarized in terms of confidence intervals and *t*-tests. Analogous models assessed the RBANS global cognitive function standardized score and subdomain standardized scores as outcomes. Effect sizes for RBANS were defined as the estimated means divided by the global reference standard deviation (i.e., 15) (Duff et al., 2008). Data were analyzed on an intention to treat basis, i.e., according to group assigned at the time of randomization regardless of adherence to the intervention. Income, education, employment didn’t differ between groups and were not included in the models as covariates. Due to prior reports that BMI may influence the exercise-cognition relationship, BMI was included as a covariate in the models (Chang et al., 2016). The alpha threshold was set at 0.05. For the final analyses, *N* = 28 was included for both randomization groups (two participants failed to return to the clinic and only had baseline data). We ran a power analysis to determine sample size for changes in step count (i.e., the primary goal of the study) (Newton et al., 2022). This power analysis showed that *N* = 28 provides 80% power to detect a 2,000 step count difference with 20% drop out and alpha = 0.05. Based on the prior literature, change in activity was considered to have a strong enough relationship with cognitive function for our analyses to detect a difference with this sample size (Etnier et al., 1997; Bherer et al., 2013).

### Secondary Analyses

Secondary analyses added gender as a predictor to the primary analysis models (*N* = 54 with complete data and *N* = 2 with just baseline data). We also investigated whether the amount of MVPA may have influenced the results. A sensitivity analysis was performed by adding MVPA change between baseline and week 12 (i.e., ≥ 5-min increase and < 5-min increase) as a covariate. Metabolic variables at baseline (i.e., glucose, total cholesterol, LDL, HDL, BMI) were separately added into the models to test their influence on the physical activity and cognition relationships. In a separate analysis, individuals who changed medication (i.e., hypertension and cholesterol medication) during the intervention were removed to control for possible medication effects.

## Results

### Participants

**FIGURE 1:**
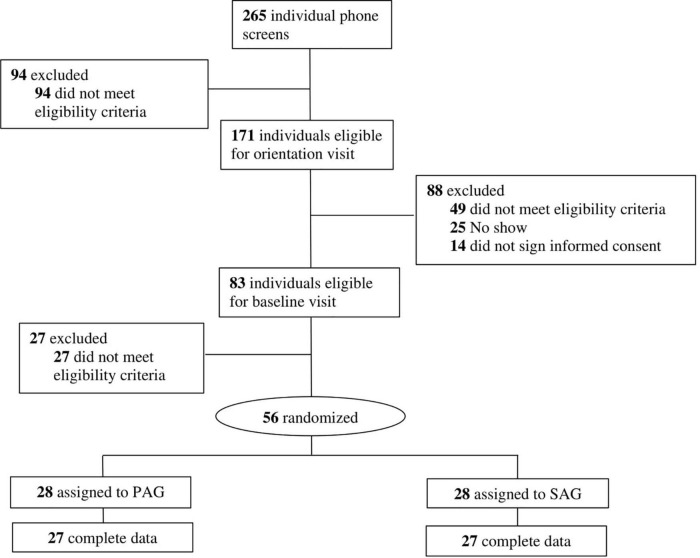
Consort flow diagram.

There were 56 participants enrolled in the PAACE study (see Figure 1). Recruitment for this study occurred between March 2018 and April 2019 with the cessation occurring when the target sample size was achieved. The mean age of the sample was 69.2 ± 3.4 years old, 73% of participants were female, and the majority were retired (75%). Most fell into the obesity Class 1 category (avg. BMI 32.5 ± 6.1), had at least 1–3 years of college education and less than $50,000 of yearly household income. SAG and PAG participants did not have significant differences between sex, age, BMI, education, and income at baseline. They engaged in 6.3 ± 6.8 min of MVPA per week at baseline and had baseline RBANS global cognitive function and subdomain scores ranging from 93.6 to 95.3 and 86.8 to 102.9, respectively (see Tables 1, 2). Participants reported no adverse events or injuries related to the study.

**TABLE 1:** Baseline participant characteristics.

	All	PA group	SA group	*P*-value
	(*n* = 56)	(*n* = 28)	(*n* = 28)	
Age, y	69.2 (3.4)	68.9 (3.8)	69.6 (2.9)	0.41
Gender, female *n*	41 (73.2%)	20 (71.4%)	21 (75.0%)	0.76
Weight, lbs.	194.1 (37.6)	202.4 (39.8)	185.8 (34.0)	0.10
BMI	32.5 (6.1)	33.8 (6.1)	31.2 (5.8)	0.11
**Employment**				
Full-time	4 (7.1%)	1 (3.6%)	3 (10.7%)	0.50
Part-time	10 (17.9%)	6 (21.4%)	4 (14.3%)	
Retired	42 (75.0%)	21 (75.0%)	21 (75.0%)	
**Education**				
Some high school				
High school diploma/GED	8 (14.3%)	6 (21.4%)	2 (7.1%)	0.21
1–3 years college	18 (32.1%)	8 (28.6%)	10 (35.7%)	
College degree	15 (26.8%)	9 (32.1%)	6 (21.4%)	
Post graduate degree	15 (26.8%)	5 (17.9%)	10 (35.7%)	
**Income**				
<$50,000	36 (64.3%)	19 (67.9%)	17 (60.7%)	0.35
50,000–100,000	14 (25.0%)	5 (17.9%)	9 (32.2%)	
>$100,000	4 (7.1%)	2 (7.1%)	2 (7.1%)	
Did not answer	2 (3.6%)	2 (7.1%)	0 (0.0%)	
**Physical activity**				
ActiGraph MVPA, min	6.3 (6.8)	7.8 (8.1)	4.8 (5)	0.10

*Each table entry shows either the number of individuals (percentage of sample) or the mean of a measurement (standard deviation).*

**TABLE 2:** RBANS total scale and subscales.

	PA				SA				Group differences over time	
			
RBANS	W0 ($\bar{x}$ ± S.E.)	W12 ($\bar{x}$ ± S.E.)	Change over time ($\bar{x}$ ± S.E.)	*P*-value	W0 ($\bar{x}$ ± S.E.)	W12 ($\bar{x}$ ± S.E.)	Change over time ($\bar{x}$ ± S.E.)	*P*-value	$\bar{x}$ ± S.E.	*P*-value
Global cognitive function	93.6 ± 2.1	94.6 ± 2.5	1.0 ± 1.7	0.57	95.29 ± 2.15	100.90 ± 2.49	5.61 ± 1.82	**0.003**	−4.6 ± 2.5	0.07
Subdomains										
Immediate memory	99.1 ± 2.8	100.1 ± 3.1	1.0 ± 2.4	0.66	98.8 ± 2.8	104.3 ± 3.1	5.43 ± 2.43	**0.03**	4.4 ± 3.4	0.20
Visuospatial	86.8 ± 2.8	87.9 ± 2.9	1.1 ± 2.4	0.65	92.4 ± 2.8	98.9 ± 2.9	6.6 ± 2.5	**0.01**	−5.5 ± 3.5	0.13
Language	98.9 ± 2.1	98.7 ± 1.8	−0.3 ± 2.0	0.90	95.9 ± 2.1	98.6 ± 1.9	2.7 ± 2.0	0.19	−2.9 ± 2.8	0.31
Attention	93.0 ± 2.7	93.3 ± 2.4	0.3 ± 2.0	0.90	91.8 ± 2.7	95.0 ± 2.5	3.3 ± 2.1	0.13	−3.0 ± 2.9	0.31
Delayed memory	99.9 ± 2.5	102.5 ± 2.9	2.6 ± 1.5	0.09	102.9 ± 2.6	106.5 ± 2.9	3.6 ± 1.6	**0.03**	−0.9 ± 2.2	0.68

*$\bar{x}$ ± S.E, mean ± standard error. Bold values have p < 0.05.*

### Attendance

Participants attended an average of 93 and 86% of their assigned PAG and SAG group sessions, respectively.

### Physical Activity

PAG participants increased minutes of daily MVPA more than SAG participants did (6.40 ± 1.67 vs. 1.72 ± 1.67 min/week; *p* = 0.02). There were no significant SAG-PAG differences in change in light or sedentary activity (*p*-values = 0.587 and *p* = 0.523). In addition, PAG participants increased steps per day significantly more than SAG participants did (1085.3 ± 265.6 vs. 34.7 ± 264.3 steps/day; *p* = 0.008).

### Repeatable Battery for the Assessment of Neuropsychological Status

**FIGURE 2:**
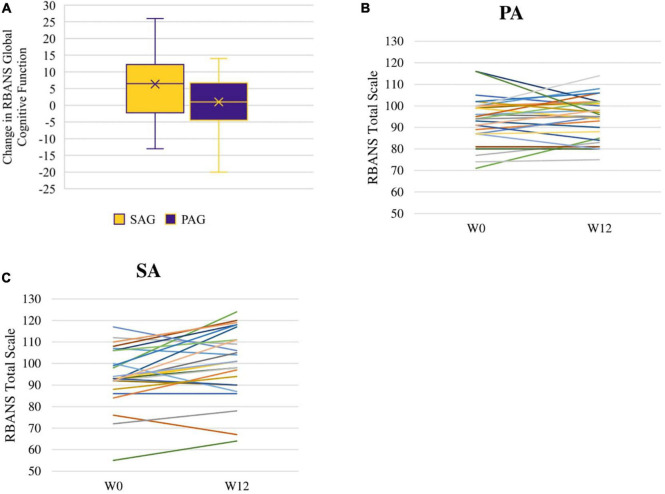
RBANS total scale change between week 0 (W0) and week 12 (W12): distribution of change within SAG and PAG groups **(A)** and spaghetti plots of individual changes among PA **(B)** and SA **(C)** participants.

**FIGURE 3:**
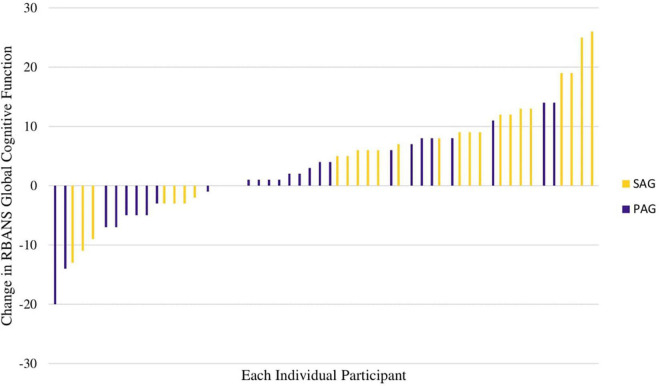
Waterfall plot of individual changes in RBANS total scale change, colored by group assignment (SAG vs. PAG).

The RBANS global cognitive function score increased more in the SAG than in the PAG [Change Est. (PAG minus SAG) -4.6 ± 2.5 points effect size = 0.31] at a trend level (*p* = 0.072). The RBANS global cognitive function score increased significantly within the SAG (Est. 5.6 ± 1.8, *p* = 0.0032, effect size = 0.37; Figure 2A). Changes in individual RBANS global cognitive function score are illustrated in Figures 2B,C, 3. SAG scores on visuospatial function (Est. 6.6 ± 2.5, effect size = 0.44), delayed memory (Est. 3.6 ± 1.6, effect size = 0.24), and immediate memory (Est. 5.4 ± 2.4, effect size = 0.36) subdomains all significantly increased as well (all *p*-values < 0.05). The RBANS global cognitive function score and subdomain scores for PAG and SAG are shown in Table 2. Females in the SAG (Est. 8.5 ± 2.0, effect size = 0.57) increased their RBANS global cognitive function more than SAG and PAG males did (SAG: Est. 8.45 ± 3.85, effect size = 0.56; PAG: Est. –10.40 ± 3.92, effect size = 0.69) and more than PAG females did (Est. –6.68 ± 2.73, effect size = 0.45; all *p*-values < 0.035). RBANS global cognitive function changes did not differ significantly between PAG males and females, nor between SAG and PAG males (see Table 3). The above results were not modified significantly when MVPA was included as a covariate (data not shown). Baseline metabolic variables were non-significant when added to the models, and the sub-sample that omitted individuals who underwent medication changes during the study had results that were similar to those of the full sample (data not shown).

**TABLE 3:** Cells along the main diagonal: within-group change over time in RBANS total scale. Off-diagonal terms: group differences in RBANS total scale.

Sex and assignment groups	PAG male	PAG female	SAG male	SAG female
PAG male	−1.92 ± 3.35			
PAG female	3.72 ± 3.87	1.80 ± 1.90		
SAG male	−1.94 ± 4.68	1.78 ± 3.81	0.02 ± 3.30	
SAG female	−10.40 ± 3.92**	−6.68 ± 2.73**	8.45 ± 3.85*	8.47 ± 1.97***

Mean ± standard deviation are shown. *p < 0.05, **p < 0.02, ***p < 0.0001.

## Discussion

In this randomized controlled trial among cognitively healthy older AAs, global cognitive function improved more within an educational control group (SAG) than it did in a PAG at a trend level. Participants in the SAG showed significant improvements in select cognitive domains. Females in the SAG may have been the primary drivers of improvement over time and differences from the PAG, as they improved significantly more than SAG males, PAG males, and PAG females. These results suggest that the participants, especially females, may have experienced more cognitive benefit from their experience in the SAG in comparison to the PAG.

To date, exercise intervention studies with a similar length and age range have either reported a lack of significant cognitive changes over time within both exercise and control groups, a lack of significant differences between exercise and control groups, or domain specific improvements within the exercise group (Emery and Gatz, 1990; Predovan et al., 2012; Esmail et al., 2020). Our study contributes to this already mixed literature by demonstrating a new pattern of results: domain-specific improvements within the control group only, and a trend level difference in global cognitive change that favors the control group. The mixed state of the literature may be due to differences in trial designs, such as different exercise dose or cognitive function assessment (Snowden et al., 2011; Northey et al., 2018). In addition, as prior trials have largely not focused on exercise’s effects on cognitive performance among older African American adults, our differing results may be due to differences in exercise responses specifically within this community.

Our results contrast with those of two recently published trials (Note: Wharton and colleagues results were an Alzheimer’s Association published poster presentation with the clinical trial design published by Hackney et al., 2019) that did focus on middle aged/older African American adults (Hackney et al., 2019; Fausto et al., 2021; Wharton et al., 2021). These interventions utilized dance-based physical activity (i.e., cardio dance routines and adapted tango) and reported domain-specific cognitive improvements over time in the exercise condition in comparison to control. Sample sizes were smaller in comparison (i.e., adapted tango *N* = 24 and cardio dance routines *N* = 32) and only one was set in the community (i.e., cardio dance routines) However, direct comparisons between trials are difficult as one trial was significantly longer (i.e., 5 months) and the other trial focused specifically on African American women with a family history of Alzheimer’s Disease. Due to the mixed nature of the results in this small base of evidence, more exercise trials focused on cognitive outcomes are needed in this population (e.g., NCT03890861, and NCT04956549).

The intervention led to a 6.4 min/day increase in MVPA (cumulatively about 45 min of MVPA weekly). Previous literature has indicated that similar changes in activity can have positive influences on body composition and cardiometabolic outcomes (Wijsman et al., 2013; Kerr et al., 2018). However, there may have been an insufficient intensity or dose of physical activity to provoke significant improvement in cognitive function in the PAG. A meta-analysis of exercise interventions for older adults concluded that exercise interventions involving a duration of 45–60 min per session, and an intensity that was at least moderate, provided cognitive improvements on average (Northey et al., 2018). A recent narrative review supported this conclusion with the caveat that physical activity at any intensity could be beneficial to older adults (Quigley et al., 2020). The PAACE intervention was designed for participants to progressively reach moderate to vigorous intensity in 30–45-min bouts. Accelerometer data show that while participants were engaging in 30 min of group-based MVPA, they only achieved 20 min of home-based MVPA (Newton et al., 2022). Therefore, participants in the intervention may not have been reaching the duration of MVPA per bout needed to stimulate positive cognitive changes.

Participants in the SAG may have experienced improved cognitive function due to behavior change in multiple domains or due to group dynamics. Approximately 31% of SAG participants (compared to 68% of PAG participants) increased time spent in MVPA more than 5 min per day, although the average MVPA change in the SAG was not statistically significant. Participants in the SAG received health information on topics such as healthy eating and sleep, yet they did not report significant dietary changes and sleep was unmeasured. SAG participants may have made subtle improvements in diet and sleep which could have contributed to cognitive improvements as both are associated with improved cognitive functioning (Dzierzewski et al., 2018; Radd-Vagenas et al., 2018). Finally, individuals in the SAG were encouraged to engage in group discussion during the sessions, creating a social environment and possibly provoking increased social engagement. Social engagement has been shown to have a positive effect on cognitive functioning in prior studies among AAs (Sims et al., 2011; Pugh et al., 2021a). Each of these changes alone is unlikely to have a major impact on cognition over 12 weeks. However, simultaneous changes across multiple behaviors (e.g., diet, physical activity, social engagement, sleep) has resulted in improvements in cognitive function in prior studies (Ngandu et al., 2015), thus supporting the concept that such multi-faceted behavior changes in SAG may have influenced our findings. Future work should determine the degree to which simultaneous modification of multiple lifestyle behaviors enhances cognitive outcomes among older AAs. To date, few of these programs targeting AAs exist (Rovner et al., 2018).

Women in the SAG had significantly greater enhancement in cognitive performance than other groups. Prior literature suggests that social engagement and support is an important component to women’s health (Fothergill et al., 2011; Johnson, 2014), and thus women in the SAG may have benefitted more from social engagement than men in the SAG. Alternatively, men are less likely to adhere to medical advice; this behavior may have prevented them from catalyzing the healthy living information given to them in the SAG sessions into healthy behavior changes (Watkins et al., 2010). Yet, it was important to note that this result needs to be interpreted with caution as the number of men in this study was small.

Although prior literature has suggested that several cardiometabolic factors (i.e., fasting plasma glucose, cholesterol, LDL, HDL, and BMI) are associated with cognitive function in older adults (Tan et al., 2011; Ma et al., 2017), our secondary analyses did not suggest that these variables influenced the relationship between physical activity and cognition. Changes to hypertension and cholesterol medications had little effect either (Schreurs, 2010; Elias et al., 2012). We speculate that longer-term follow-up may be needed with deeper characterization of cardiometabolic factors, to detect effects that cardiometabolic factors may have had on intervention responses.

This study had some important strengths. This study had a randomized control trial design and it was informed by focus groups of its target population (i.e., older AAs) (Pugh et al., 2021b). However, a limitation was that the study was only 12 weeks in duration. Additionally, there was a small amount of African American men although the male subsample was evenly distributed between intervention groups. In conclusion, individuals receiving health education enhanced their cognitive function over the course of the 12-week program. Future research should focus on improving the effectiveness of the PA program, investigate what components made this successful aging program effective and why women seemed to benefit the most.

## Data Availability Statement

The raw data supporting the conclusions of this article will be made available by the authors, without undue reservation.

## Ethics Statement

The studies involving human participants were reviewed and approved by the Pennington Biomedical Research Center Institutional Review Board. The patients/participants provided their written informed consent to participate in this study.

## Author Contributions

RN and OC contributed to the conception, design, and execution of the study. RBr, RBe, WJ, CH, LC, and MH contributed to the execution and some design of the study. WJ and RBe organized the database and performed the statistical analysis. KG wrote the first draft of the manuscript and collaborated with RBe for the direction of the statistical analyses. All authors contributed to manuscript revision, read, and approved the submitted version.

## Conflict of Interest

The authors declare that the research was conducted in the absence of any commercial or financial relationships that could be construed as a potential conflict of interest.

## Publisher’s Note

All claims expressed in this article are solely those of the authors and do not necessarily represent those of their affiliated organizations, or those of the publisher, the editors and the reviewers. Any product that may be evaluated in this article, or claim that may be made by its manufacturer, is not guaranteed or endorsed by the publisher.
